# Dietary phosphorus intake modifies the association between total cholesterol and lumbar spine bone mineral density: results from NHANES 2011–2016

**DOI:** 10.3389/fnut.2025.1509287

**Published:** 2025-03-28

**Authors:** Dechen Yu, Pan Li, Kangkang Su, Xiongfei Cao, Xiaolei Yu, Zhengxu Ye, Mo Li

**Affiliations:** ^1^Department of Orthopedics, The First Affiliated Hospital of Air Force Medical University, Xi'an, China; ^2^Department of Cardiology, Air Force Medical University Tangdu Hospital, Xi'an, China

**Keywords:** dietary phosphorus intake, total cholesterol, lumbar spine bone mineral density, interaction, cross-sectional study

## Abstract

**Background:**

The connection between total cholesterol (TC) and lumbar spine bone mineral density (BMD) is well-documented, yet the role of dietary phosphorus intake in this relationship is not fully understood. This cross-sectional study aims to explore how dietary phosphorus affects the link between TC and lumbar spine BMD.

**Methods:**

Data from the National Health and Nutrition Examination Survey (NHANES) spanning 2011 to 2016 were analyzed, involving 7,155 participants. Based on the median daily phosphorus intake, participants were divided into a low phosphorus intake group (phosphorus intake <1,445 mg/d) and a high phosphorus intake group (phosphorus intake ≥ 1,445 mg/d). A multiple linear regression analysis was performed to investigate the association between TC and lumbar spine BMD, with a focus on determining if dietary phosphorus intake may serve as a potential influencing factor.

**Results:**

The study revealed a negative association between TC and lumbar spine BMD. The strength of this relationship varied between the low and high phosphorus intake groups, with *β* values of −0.219 (95% CI: −0.334 to −0.105) for the low group and − 0.420 (95% CI: −0.548 to −0.291) for the high group. Additionally, there was an interaction between total cholesterol and dietary phosphorus intake in reducing lumbar spine bone density (*P* for interaction = 0.0168).

**Conclusion:**

Our study results indicate that dietary phosphorus intake influences the relationship between TC and lumbar spine BMD, which may have important implications for clinical management.

## 1 Introduction

Osteoporosis is a metabolic disease characterized by decreased bone density (1). With the increasing aging population worldwide, the prevalence of osteoporosis is rising annually, making it a global public health concern (2). Understanding the pathogenesis of osteoporosis is essential for its prevention.

Elevated total cholesterol (TC) is becoming an increasingly common health issue. It is well known that total cholesterol is related to lipid metabolism, and furthermore, it can promote atherosclerosis, leading to cardiovascular and cerebrovascular diseases (3). However, the relationship between TC and lumbar spine bone mineral density (BMD) remains controversial. Zhang et al. found a non-linear relationship between total cholesterol and lumbar spine BMD: when TC is below 5.86 mmol/L, there is a negative correlation, but when TC exceeds 5.86 mmol/L, the relationship is reversed (4). Jeong et al. reported that as TC increases, lumbar spine BMD also increases (5). Additionally, other studies have shown no correlation between TC and lumbar spine BMD (6). The discrepancies in these findings may be due to the failure to account for potential confounding factors, such as dietary phosphorus intake.

Phosphorus plays a crucial role in the processes of bone resorption and dissolution (7). Previous research has indicated that a high intake of phosphorus contributes positively to the maintenance of lumbar spine bone mineral density (BMD) (8). Based on previous experience (9), “high phosphorus intake” in our study was defined as ≥1,445 mg/ day (median for NHANES participants), consistent with previous reports of dietary phosphorus exceeding the recommended daily intake (700 mg/day for Institute of Medicine adults). High phosphorus intake may impair BMD through multiple pathways: (1) Osteoblast Suppression: Elevated serum phosphate inhibits osteoblast differentiation via downregulation of Runx2 and Osterix (7); (2) Osteoclast Activation: Phosphate stimulates RANKL expression, promoting osteoclast-mediated bone resorption (10); (3) Calcium-Phosphorus Imbalance: Excess phosphorus activates the FGF23-Klotho axis, reducing bioactive vitamin D and impairing calcium absorption, exacerbating demineralization (11); (4) Inflammatory Synergy: Chronic inflammation (e.g., from hypercholesterolemia) amplifies phosphate-induced IL-6 and TNF-*α*, which further destabilizes bone remodeling (12). Another study demonstrated that a diet rich in phosphorus could lower cholesterol levels in both the serum and tissues of weaned hybrid pigs (13). Elevated TC has been associated with reduced bone turnover markers and altered osteoblast function, suggesting a potential mechanistic link between lipid metabolism and bone health (14). However, few experiments have explored the impact of phosphorus intake on the relationship between TC and lumbar spine BMD. In this cross-sectional study, we propose the hypothesis that phosphorus and TC may have an interactive effect on lumbar spine BMD. Our aim is to investigate the influence of phosphorus intake on the relationship between TC and lumbar spine BMD.

## 2 Methods

### 2.1 Data source

This study utilized data from the National Health and Nutrition Examination Survey (NHANES) for the years 2011–2016. NHANES is a cross-sectional survey focused on diet and health among non-institutionalized residents of the United States. The data collected include demographic information, dietary intake, questionnaires, laboratory data, and physical measurements, using a multistage stratified probability design. Health interviews took place in the participants’ homes, whereas extensive physical examinations, which included blood sample collection, were conducted at mobile testing centers. Serum samples were subsequently analyzed in laboratories. All study protocols were approved by the NCHS Research Ethics Review Board, and all participants provided written informed consent^1^ (15).

### 2.2 Measurement of TC

Analyze serum TC levels in venous samples according to the standardized protocol and measure them using an enzyme-coupled reaction. Specific information can be obtained from the NHANES official website.

### 2.3 Phosphorus intake

The data on dietary phosphorus in this study was determined through a 24-h dietary recall. This approach is the most widely utilized in large-scale surveys to evaluate dietary intake. This protocol is based on a consensus reached during an expert assessment workshop conducted as part of the NHANES. Phosphorus intake was classified as high or low based on the median daily intake of 1,445 mg.

### 2.4 Measurement of lumbar spine BMD

Lumbar spine BMD, the primary outcome variable, was obtained through Dual-energy x-ray absorptiometry (DXA) scans performed by trained and certified radiologic technologists. All scans were conducted on a Hologic Discovery A densitometer (Hologic, Inc., Bedford, Massachusetts) using Apex 3.2 software version, and lumbar spine bone density was reported in gm/cm2. Detailed information regarding the lumbar spine bone density examination is documented in the “Body Composition Procedures Manual” on the NHANES website.

### 2.5 Covariates

In this study, age, gender, race, education level, smoking, family income, BMI, diabetes, hypertension, moderate physical activity and biochemical indicators (blood urea nitrogen, total calcium, total protein, phosphorus, uric acid and direct HDL-cholesterol) were taken as potential regulatory covariates in the research model. The race categories consist of Mexican Americans, other Hispanic, Non-Hispanic White, Non-Hispanic Black, and other races. Education level was divided into below high school, high school graduate, and college or higher. Smoking status was determined by whether participants had smoked over 100 cigarettes in their lifetime, classifying them as smokers or non-smokers. Diabetes, hypertension, and physical activity were determined based on self-reported information from participants. Household income was assessed through the poverty-income ratio (PIR), which takes family size into account. BMI was calculated based on the participant’s height and weight. Additionally, specific information regarding various biochemical indicators (blood urea nitrogen, total calcium, total protein, phosphorus, uric acid and direct HDL-cholesterol) were sourced from NHANES laboratory results.

### 2.6 Statistical analysis

All statistical analyses were performed using EmpowerStats software^2^ and R software. To investigate the relationship between total cholesterol and lumbar spine bone density, we conducted a multiple linear regression. Lumbar spine bone density was evaluated across different levels of phosphorus intake, and intergroup interactions were assessed using likelihood ratio tests. Additionally, 95% confidence intervals (CIs) were calculated, with differences considered clinically significant if *P* < 0.05. In descriptive analysis, continuous variables were presented as mean and standard deviation (SD) or median and interquartile range (IQR). Categorical variables were expressed as weighted percentages (%). Chi-square tests (for categorical variables) and t-tests (for normally distributed variables) or Kruskal-Wallis tests (for skewed variables) were used to compare continuous and categorical variables, respectively.

## 3 Results

### 3.1 Selection of participants and basic characteristics

**Figure 1:**
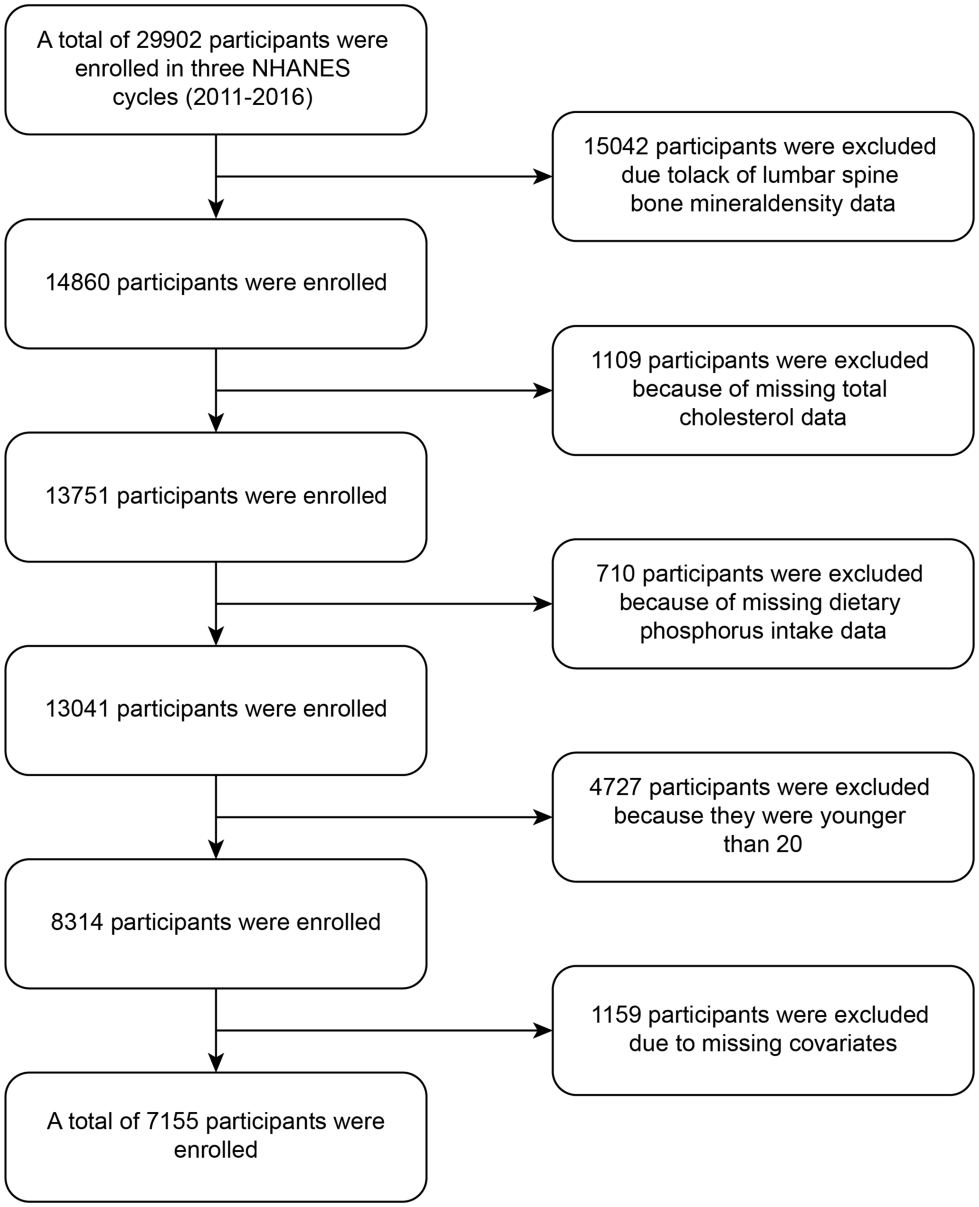
Flowchart of participants enrollment.

This study utilized data from three NHANES cycles: 2011–2012, 2013–2014, and 2015–2016. The flowchart for the participant selection process is shown in Figure 1. Out of 29,902 participants, we excluded those without lumbar spine BMD measurements (*n* = 15,402), TC data (*n* = 1,109), and dietary phosphorus intake data (*n* = 710), as well as individuals under 20 years of age (*n* = 4,727) and those with missing covariate information (*n* = 1,159). Ultimately, a total of 7,155 individuals were included in the final analysis. Based on their dietary phosphorus intake, participants were divided into two groups, with their basic characteristics presented in Table 1. The high-phosphorus group included 11.6% Mexican American, 10.0% Other Hispanic, 36.5% Non-Hispanic White, 24.2% Non-Hispanic Black, and 17.7% Other Races. The low-phosphorus group included 16.3% Mexican American, 9.2% Other Hispanic, 42.5% Non-Hispanic White, 17.9% Non-Hispanic Black, and 14.1% Other Races. The results revealed significant differences (*P*-value <0.05) between the low-phosphorus and high-phosphorus intake groups in terms of age, gender, race, PIR, alcohol consumption, moderate physical activity, blood urea nitrogen, total calcium, phosphorus, uric acid, direct high-density lipoprotein, and lumbar spine BMD.

**Table 1:** Baseline characteristics of participants.

Covariates	Dietary phosphorus intake (mg/d)		*P*-value
	<1,445 mg/d (*n* = 4,138)	≥ 1,445 mg/d (*n* = 3,017)	
Age (years, mean ± SD)	39.4 ± 11.7	38.6 ± 11.4	0.008
Gender, *n* (%)			<0.001
Male	1700 (41.1%)	2004 (66.4%)	
Female	2,438 (58.9%)	1,013 (33.6%)	
Race, *n* (%)			<0.001
Mexican America	481 (11.6%)	492 (16.3%)	
Other Hispanic	415 (10.0%)	277 (9.2%)	
Non-Hispanic White	1,511 (36.5%)	1,283 (42.5%)	
Non-Hispanic Black	1,000 (24.2%)	540 (17.9%)	
Other races	731 (17.7%)	425 (14.1%)	
Education, *n* (%)			0.518
Under high school	683 (16.5%)	469 (15.5%)	
High school or equivalent	902 (21.8%)	655 (21.7%)	
Above high school	2,553 (61.7%)	1893 (62.7%)	
PIR (mean ± SD)	2.5 ± 1.6	2.6 ± 1.7	<0.001
BMI (kg/m^2^, Mean ± SD)	28.6 ± 6.7	28.6 ± 6.6	0.904
Smoked at least 100 cigarettes in life, *n* (%)			0.171
Yes	1,652 (39.9%)	1,253 (41.5%)	
No	2,486 (60.1%)	1764 (58.5%)	
Had at least 12 alcohol drinks past 1 year? *n* (%)			<0.001
Yes	3,031 (73.2%)	2,448 (81.1%)	
No	1,107 (26.8%)	569 (18.9%)	
Diabetes, *n* (%)			0.557
Yes	326 (7.9%)	219 (7.3%)	
No	3,735 (90.3%)	2,746 (91.0%)	
Borderline	77 (1.9%)	52 (1.7%)	
Hypertension, *n* (%)			0.960
Yes	1,002 (24.2%)	729 (24.2%)	
No	3,136 (75.8%)	2,288 (75.8%)	
Moderate work activity, *n* (%)			<0.001
Yes	1,586 (38.3%)	1,305 (43.3%)	
No	2,552 (61.7%)	1712 (56.7%)	
Blood urea nitrogen (mg/dL, mean ± SD)	11.8 ± 4.3	13.0 ± 4.5	<0.001
Total calcium (mg/dL, mean ± SD)	9.4 ± 0.3	9.4 ± 0.3	0.002
Phosphorus (mg/dL, mean ± SD)	3.7 ± 0.6	3.8 ± 0.6	<0.001
Total protein (g/dL, mean ± SD)	7.2 ± 0.5	7.2 ± 0.4	0.780
Uric acid (mg/dL, mean ± SD)	5.3 ± 1.4	5. 5 ± 1.4	<0.001
Direct HDL-Cholesterol (mg/dL, mean ± SD)	52.7 ± 15.4	50.8 ± 15.3	<0.001
Total cholesterol (g/dL, mean ± SD)	0.2 ± 0.0	0.2 ± 0.0	0.162
Lumbar spine BMD (g/cm^2^, mean ± SD)	1.0 ± 0.2	1.0 ± 0.2	0.002

### 3.2 Association between TC and lumbar spine BMD

Table 2 presents the association between TC and lumbar spine BMD. In the unadjusted analysis (Model 1), a negative correlation was observed between TC and lumbar spine BMD. Similar results were observed in the minimally adjusted model (Model 2) and the fully adjusted model (Model 3) [−0.310 (−0.399, −0.220)]. Furthermore, when TC was converted into a categorical variable, the trend test remained significant (*P* < 0.001). The negative correlation between TC and lumbar spine bone density was validated in the minimally adjusted model of KNHANES database (Supplementary Table S1).

**Table 2:** Association between TC and lumbar spine BMD.

	Model 1 *β* (95% CI) *P* value	Model 2 *β* (95% CI) *P* value	Model 3 *β* (95% CI) *P* value
Total cholesterol (g/dL)	−0.401 (−0.485, −0.317) <0.00001	−0.292 (−0.378, −0.206) <0.00001	−0.310 (−0.399, −0.220) <0.00001
Total cholesterol categories			
Q1 (0.059–0.162 g/dL)	Reference	Reference	Reference
Q2 (0.163–0.186 g/dL)	−0.016 (−0.026, −0.006) 0.00130	−0.010 (−0.020, −0.000) 0.04162	−0.011 (−0.021, −0.001) 0.02641
Q3 (0.187–0.213 g/dL)	−0.017 (−0.027, −0.007) 0.00081	−0.009 (−0.018, 0.001) 0.08569	−0.010 (−0.020, −0.000) 0.04063
Q4 (0.214–0.545 g/dL)	−0.044 (−0.054, −0.035) <0.00001	−0.032 (−0.042, −0.022) <0.00001	−0.034 (−0.044, −0.023) <0.00001
*P* for trend	<0.001	<0.001	<0.001

### 3.3 The impact of dietary phosphorus intake on the relationship between total cholesterol and lumbar spine bone mineral density

The univariate analysis indicated that age, PIR, BMI, Diabetes, HDL-Cholesterol, and TC were associated with lumbar spine BMD (Supplementary Table S2). Table 3 details the influence of dietary phosphorus intake on the correlation between TC and lumbar spine BMD. The results across Model 1, 2, and 3 were consistent, showing a significant negative correlation between TC and lumbar spine BMD in both the high-phosphorus and low-phosphorus dietary groups. In Model 3, the *β* value for the high phosphorus intake group was −0.420, which was lower compared to the low phosphorus intake group with a β value of −0.219. The interaction effect between phosphorus intake and TC on lumbar spine BMD was statistically significant (*P* for interaction = 0.0168). The source of dietary phosphorus may play a significant role in its effect on lumbar spine BMD. To address this, we have conducted additional analyses to stratify the high phosphorus intake group based on calcium intake levels. The Supplementary Table S3 showed that the negative association between TC and lumbar spine BMD was more pronounced in the high phosphorus/low calcium subgroup compared to the high phosphorus/high calcium subgroup (−0.124 vs. −0.149). This suggests that the interaction between phosphorus and calcium intake may modulate the relationship between TC and lumbar spine BMD. The *β* values and 95% CI of confounders in the linear regression model are shown in Supplementary Table S4. The variance inflation factors (VIF) of all variables are shown in Supplementary Table S5, and VIF is less than 2.0, indicating that there is no significant multicollinearity in the model.

**Table 3:** Interactive effect of TC and dietary phosphorus intake on lumbar spine BMD.

	Model 1 *β* (95% CI) *P* value	Model 2 *β* (95% CI) *P* value	Model 3 *β* (95% CI) *P* value
Low-phosphorus intake (<1,445 mg/d, *n* = 4,138)	−0.318 (−0.429, −0.207) <0.0001	−0.195 (−0.308, −0.082) 0.0007	−0.219 (−0.334, −0.105) 0.0002
High-phosphorus intake (≥ 1,445 mg/d, *n* = 3,017)	−0.504 (−0.631, −0.377) <0.0001	−0.407 (−0.534, −0.280) <0.0001	−0.420 (−0.548, −0.291) <0.0001
*P* for interaction	0.0305	0.0119	0.0168

## 4 Discussion

This study analyzed a nationally representative sample and found that total cholesterol was negatively associated with lumbar spine bone density. In addition, dietary phosphorus intake and total cholesterol interacted to reduce lumbar spine bone density in most models. Specifically, the negative association between TC and BMD was stronger in the high-phosphorus intake group (*β* = −0.420) compared to the low-phosphorus group (β = −0.219). This suggests that elevated phosphorus intake exacerbates the adverse effects of TC on bone density, potentially through mechanisms such as calcium-phosphorus imbalance and increased bone resorption (Table 3).

Lipid metabolism is involved in the pathogenesis of osteoporosis (16, 17). TC plays a pivotal role in this process, as both cholesterol and its metabolites can inhibit osteoblast differentiation (18). Furthermore, TC serves as a precursor for vitamin D synthesis. Given that vitamin D is essential for calcium absorption and bone health, fluctuations in cholesterol levels may impact vitamin D levels, thereby influencing lumbar spine BMD (19, 20). Consequently, lumbar spine BMD may be affected by TC either directly or indirectly through these mechanisms. Consistent with our findings, Fang et al. reported a negative correlation between serum TC and lumbar spine BMD in American women over 45 years of age, with a more pronounced association observed in those with a BMI below 24.9 kg/m^2^ (21). Similarly, Hu et al. arrived at the same conclusion in their study (22). Additionally, a meta-analysis of 33 studies indicated that statin use can increase BMD in both the lumbar spine and hip, while also reducing the overall risk of fractures and hip fractures (23). Nevertheless, the relationship between serum total cholesterol and lumbar spine BMD remains contentious. A cohort study involving 289 male patients identified a positive correlation between serum TC and BMD in both the lumbar spine and hip (24). Thus, further research is warranted to elucidate the role of serum TC in lumbar spine BMD.

In addition, it is important to differentiate between serum lipids and bone marrow fat, two biologically distinct compartments with unique roles in bone metabolism. While bone marrow adiposity is well-established as a negative correlate of BMD (25, 26), the relationship between serum cholesterol and BMD remains less definitive. Serum lipids, such as LDL-C and HDL-C, may influence bone health through divergent pathways. For instance, LDL-C has been associated with reduced lumbar BMD in women, whereas HDL-C—often considered cardioprotective—may exert positive effects on skeletal integrity (27, 28). These findings underscore the complexity of lipid-BMD interactions and highlight the need to evaluate lipoprotein subfractions independently. Emerging evidence suggests that lipoprotein subfractions may modulate BMD through distinct mechanisms. LDL-C, a key driver of atherosclerosis, may promote oxidative stress and inflammation, indirectly accelerating bone resorption. Conversely, HDL-C—via its anti-inflammatory and antioxidant properties—could mitigate these effects, potentially preserving bone density (27). Our study focused on TC as a composite measure, but future investigations should explore subtype-specific associations to refine clinical interpretations.

Phosphorus, as a key dietary mineral, interacts with lipid metabolism and may modify how cholesterol affects bone density. One possible pathway involves the role of phosphorus in lipid absorption and metabolism (29). Phosphorus, particularly in the form of phospholipids, is essential for the structural integrity of cell membranes and is involved in lipid transport mechanisms (30). High phosphorus intake, especially from inorganic sources such as phosphate additives, may alter lipid metabolism, potentially influencing the way total cholesterol levels impact bone health (31).

Accumulating evidence highlights the dual role of dietary phosphorus in bone health. While phosphorus is essential for hydroxyapatite formation, excessive intake--particularly from processed foods rich in inorganic phosphate additives--has been implicated in osteoporosis pathogenesis. Large-scale cohort studies report that individuals in the highest quintile of phosphorus intake (≥1,400 mg/day) exhibit a 1.33-fold increased risk of hip fracture compared to those with balanced intake (700–1,000 mg/day) (32). Mechanistically, our findings align with experimental models showing chronic high phosphorus: firstly, promotes pyperparathyroidism: elevated serum phosphate indirectly stimulates parathyroid hormone secretion via calcium-phosphate complex formation, accelerating bone resorption (33). Secondly, represses bone formation: excess phosphorus downregulates Wnt/*β*-catenin signaling in osteoblasts by upregulating sclerostin expression, a key inhibitor of bone formation (34).

Moreover, phosphorus is closely tied to calcium metabolism, and an imbalance in dietary phosphorus and calcium can affect bone mineralization (11). High cholesterol levels, which are often seen in conjunction with metabolic syndrome, may exacerbate this imbalance by influencing how the body metabolizes and stores calcium and phosphorus (35). For instance, some studies suggest that elevated cholesterol may impair calcium absorption in the gut, leading to increased reliance on bone stores to maintain calcium homeostasis (36). In such cases, dietary phosphorus, if consumed in excess, might accelerate bone resorption by promoting parathyroid hormone (PTH) secretion, further weakening bone density (37).

Additionally, the inflammatory processes triggered by high cholesterol levels may also interact with phosphorus to affect bone health (12, 38). Chronic inflammation has been linked to increased bone turnover and reduced bone formation (39). In such conditions, phosphorus intake may act as a modulator of these processes, influencing the inflammatory response and the subsequent impact on bone density. While adequate phosphorus is necessary for bone development, excessive intake in the presence of elevated cholesterol and inflammation may worsen bone loss (40, 41). The interaction between dietary phosphorus and total cholesterol underscores the complexity of nutrient interactions in bone metabolism. Our results indicate that the combined effect of high cholesterol and high phosphorus intake on lumbar spine bone density is greater than the sum of their individual effects. This finding emphasizes the necessity of considering dietary factors collectively when evaluating their impact on bone health, rather than in isolation.

However, this study also has certain limitations. First, given the nature of cross-sectional studies, this research cannot establish a causal relationship between dietary phosphorus intake and total cholesterol and lumbar spine bone density, which would require further cohort studies. Second, the 24-h dietary review may not reflect habitual intake and relies on memory, which is subject to some error. Third, while DEXA is the gold standard for BMD assessment, it is important to note that X-ray attenuation is influenced not only by bone mineral content but also by marrow fat. Higher marrow fat content may artifactually elevate BMD values (42). Although NHANES protocols standardize DEXA procedures, residual confounding from marrow fat cannot be entirely ruled out. Finally, although some covariates were adjusted for, residual confounding from unmeasured factors cannot be completely ruled out.

## 5 Conclusion

In conclusion, our study suggests that dietary phosphorus intake plays a mediating role in the relationship between TC and lumbar spine BMD. This finding has important clinical implications, as it highlights the need for dietary considerations in the management of osteoporosis and related conditions. Future research should aim to explore the underlying mechanisms by which phosphorus influences the effects of cholesterol on bone health, which could lead to more effective dietary recommendations and interventions for individuals at risk of osteoporosis.

## Data Availability

The raw data supporting the conclusions of this article will be made available by the authors, without undue reservation.

## Footnotes

^1^ https://wwwn.cdc.gov/nchs/nhanes/default.aspx

^2^ www.EmpowerStats.com
