# Supplementary material for: Dietary phosphorus intake modifies the association between total cholesterol and lumbar spine bone mineral density: results from NHANES 2011–2016
Source: Front Nutr. 2025 Mar 28;12:1509287. doi: 10.3389/fnut.2025.1509287 (PMC11987324; doi:10.3389/fnut.2025.1509287)
Supplement: Supplementary file 1 [file Table_1.doc]

Table S1 The relationship between TC and lumbar spine BMD in KNHANES database

|  | ***β* (95% CI)** | ***P* value** |
| --- | --- | --- |
| **TC** | -0.031(-0.200, -0.066) | <0.001 |
