# Supplementary material for: Dietary phosphorus intake modifies the association between total cholesterol and lumbar spine bone mineral density: results from NHANES 2011–2016
Source: Front Nutr. 2025 Mar 28;12:1509287. doi: 10.3389/fnut.2025.1509287 (PMC11987324; doi:10.3389/fnut.2025.1509287)
Supplement: Supplementary file 2 [file Table_2.docx]

Table S2 Association of covariates and lumbar spine BMD

| Covariates |  | P-value |
| --- | --- | --- |
| Age | 39.05±11.54 | <0.001 |
| Gender, n (%) |  | 0.604 |
| Male | 3704(51.8) |  |
| Female | 3451(48.2) |  |
| Race, n (%) |  | 0.06 |
| Mexican America | 973(13.6) |  |
| Other Hispanic | 692(9.7) |  |
| Non-Hispanic white | 2794(39) |  |
| Non-Hispanic black | 1540(21.5) |  |
| Other races | 1156(16.2) |  |
| Education, n (%) |  | 0.172 |
| Under high school | 1152(51.8) |  |
| High school or equivalent | 1557(21.8) |  |
| Above high school | 4446(62.1) |  |
| PIR | 2.54±1.66 | <0.001 |
| BMI (kg/m2) | 28.57±0.08 | <0.001 |
| Smoked at least 100 cigarettes in life, n (%) |  | 0.151 |
| Yes | 2905(40.6) |  |
| No | 4250(59.4) |  |
| Had at least 12 alcohol drinks past 1 year? n (%) |  | 0.706 |
| Yes | 5479(76.6) |  |
| No | 1676(23.4) |  |
| Diabetes, n (%) |  | 0.001 |
| Yes | 545(7.6) |  |
| No | 6481(90.6) |  |
| Borderline | 129(1.8) |  |
| Hypertension, n (%) |  | 0.06 |
| Yes | 1731(24.2) |  |
| No | 5424(75.8) |  |
| Moderate work activity, n (%) |  | 0.456 |
| Yes | 2891(40.4) |  |
| No | 4264(59.6) |  |
| Blood urea nitrogen (mg/dL) | 12.32±0.05 | 0.841 |
| Total calcium (mg/dL) | 9.39±0.004 | 0.307 |
| Phosphorus (mg/dL) | 3.75±0.07 | 0.140 |
| Total protein (g/dL) | 7.178±0.01 | 0.775 |
| Uric acid (mg/dL) | 5.39±0.02 | 0.873 |
| Direct HDL-Cholesterol (mg/dL) | 51.91±0.182 | <0.001 |
| Total cholesterol (g/dL) | 0.19±0.0005 | <0.001 |
