# Supplementary material for: Dietary phosphorus intake modifies the association between total cholesterol and lumbar spine bone mineral density: results from NHANES 2011–2016
Source: Front Nutr. 2025 Mar 28;12:1509287. doi: 10.3389/fnut.2025.1509287 (PMC11987324; doi:10.3389/fnut.2025.1509287)
Supplement: Supplementary file 3 [file Table_3.docx]

Table S3 The effect of dietary calcium on the relationship between TC and lumbar spine BMD in patients with high phosphorus intake.

|  | Model 1  β (95% CI) P value | Model 2  β (95% CI) P value | Model 3  β (95% CI) P value |
| --- | --- | --- | --- |
| Low-calcium intake  (<851 mg/d, n=506) | -0.194 (-1.116, -0.432) <0.0001 | -0.175 (-1.064, -0.347) <0.0001 | -0.149 (-0.964, -0.227) 0.002 |
| High-calcium intake  (>=851 mg/d, n=2511) | -0.130 (-0.662, -0.358) <0.0001 | -0.112 (-0.594, -0.281) <0.0001 | -0.124 (-0.644, -0.326) <0.0001 |
