# Supplementary material for: Dietary phosphorus intake modifies the association between total cholesterol and lumbar spine bone mineral density: results from NHANES 2011–2016
Source: Front Nutr. 2025 Mar 28;12:1509287. doi: 10.3389/fnut.2025.1509287 (PMC11987324; doi:10.3389/fnut.2025.1509287)
Supplement: Supplementary file 4 [file Table_4.docx]

| Variable | *β* (95% CI) | P-value |
| --- | --- | --- |
| Age | -0.116 (-0.002, -0.001) | ＜0.001 |
| Gender | -0.024(-0.016, 0.001) | 0.102 |
| Race | 0.121 (0.012, 0.018) | ＜0.001 |
| Education | 0.026(0.001, 0.011) | 0.042 |
| PIR | 0.032(0.001, 0.005) | 0.015 |
| BMI | 0.111(0.002, 0.003) | ＜0.001 |
| Smoked at least 100 cigarettes in life | 0.019(-0.002, 0.014) | 0.127 |
| Had at least 12 alcohol drinks past 1 year | -0.061(-0.031, -0.013) | ＜0.001 |
| Diabetes | -0.036(-0.030, -0.007) | 0.002 |
| Hypertension | -0.050(-0.027, -0.009) | ＜0.001 |
| Moderate work activity | -0.028(-0.016, -0.002) | 0.016 |
| Blood urea nitrogen | 0.026(0.001, 0.002) | 0.039 |
| Total calcium | -0.006(-0.014, 0.009) | 0.619 |
| Phosphorus | -0.022(-0.012, 0.001) | 0.064 |
| Total protein | 0.006(-0.006, 0.010) | 0.630 |
| Uric acid | -0.024(-0.006, 0.001) | 0.110 |
| Direct HDL-Cholesterol | 0.097(0.001, 0.001) | ＜0.001 |
| Total cholesterol | -0.100(-0.476, -0.289) | ＜0.001 |

Table S4 β values and 95% CI of confounders in linear regression models
