# Supplementary material for: Dietary phosphorus intake modifies the association between total cholesterol and lumbar spine bone mineral density: results from NHANES 2011–2016
Source: Front Nutr. 2025 Mar 28;12:1509287. doi: 10.3389/fnut.2025.1509287 (PMC11987324; doi:10.3389/fnut.2025.1509287)
Supplement: Supplementary file 5 [file Table_5.docx]

| Variable | VIF |
| --- | --- |
| Age | 1.393 |
| Gender | 1.596 |
| Race | 1.142 |
| Education | 1.288 |
| PIR | 1.289 |
| BMI | 1.379 |
| Smoked at least 100 cigarettes in life | 1.212 |
| Had at least 12 alcohol drinks past 1 year | 1.174 |
| Diabetes | 1.073 |
| Hypertension | 1.230 |
| Moderate work activity | 1.032 |
| Blood urea nitrogen | 1.178 |
| Total calcium | 1.266 |
| Phosphorus | 1.089 |
| Total protein | 1.204 |
| Uric acid | 1.648 |
| Direct HDL-Cholesterol | 1.299 |

Table S5 Variance inflation factor (VIF) for all variables
